# Overexpression of claudin-4 may be involved in endometrial tumorigenesis

**DOI:** 10.3892/ol.2013.1198

**Published:** 2013-02-18

**Authors:** XIAO-YU PAN, XUE LI, YAN-CI CHE, HONG-YAN LI, XIN LI, YUN ZHANG, XIN YANG

**Affiliations:** 1 Department of Obstetrics and Gynecology, China-Japan Friendship Hospital, Beijing 100029;; 2 Department of Pathology, Beijing Chao Yang Hospital of Capital Medical University, Beijing 100020;; 3 Department of Obstetrics and Gynecology, The Affiliated Hospital of QingDao University Medical School, QingDao 266003;; 4 Institute of Clinical Medicine, China-Japan Friendship Hospital, Beijing 100029, P.R. China

**Keywords:** endometrioid endometrial carcinoma, claudin-4, Ishikawa

## Abstract

To clarify the role of claudin-4 in endometrial tumorigenesis and to explore whether claudin-4 could be a potentially useful agent in the treatment of endometrial carcinoma, the expression of claudin-4 in endometrial carcinoma was investigated. The relationship between therapy with anti-neoplastic agents and the expression of claudin-4 was also analyzed using an endometrial carcinoma xenograft model. The expression of claudin-4 in endometrial endometrioid adenocarcinoma (EEC) and normal human endometrial tissue was determined using immunohistochemistry and real-time PCR. Ninety female BALB/c nu/nu mice were transplanted with Ishikawa endometrial cancer cells. The mice were divided into three groups with different intraperitoneal treatments: cisplatin, paclitaxel or saline solution. After the observation period tumors were extracted and stained with monoclonal antibody against claudin-4. The mRNA expression of claudin-4 was also detected using real-time PCR. Expression of claudin-4 was significantly increased at both protein and mRNA levels in the EEC group compared with the group of normal cyclic endometrium. In the study of Ishikawa xenografts, no significant changes in tumor volume and claudin-4 expression were shown in the paclitaxel group compared with the control group. A significant reduction of tumor growth and a significant decrease in claudin-4 expression were observed in the cisplatin group. These results demonstrate that claudin-4 is strongly expressed in EEC. Claudin-4 is a useful biomarker in the treatment of patients with endometrial carcinoma.

## Introduction

Endometrial carcinoma is the most common malignancy of the female genital tract. Endometrial endometrioid adenocarcinoma (EEC) accounts for ∼80% of endometrial tumors (1). To date, the etiology of endometrial carcinoma is not fully understood, although there is evidence that endocrine and genetic factors contribute to its initiation and progression (2,3).

The claudin family of proteins, the main transmembrane proteins of tight junctions, has crucial roles in the control of paracellular transport and maintenance of cell polarity (4). The recently described claudins have shown that claudins’ gene expression are frequently altered in various cancers, including gynecological cancers. Abnormal expression of claudin molecules, such as claudin-4, by neoplastic cells is possibly an important determinant of local invasion and dissemination and claudin represents a promising target for cancer detection, diagnosis and therapy (5–7). Recently, gene expression studies of primary uterine serous papillary cancer (USPC) have demonstrated that claudin-4 is one of the most highly upregulated genes in USPC when compared to normal endometrial cells (8). To determine whether claudin-4 expression has a crucial role in tumor progression, the expression of claudin-4 in EEC was investigated. To explore whether claudin-4 could be a potentially useful agent in the treatment of endometrial cancer, human endometrial cancer xenograft models were prepared and the change in claudin-4 expression in Ishikawa xenografts after treatment with cytotoxic drugs was evaluated.

## Materials and methods

### Tissue samples

Cancerous endometrium was obtained from 62 females with EEC. The tumors had been graded and staged following the current recommendations of the International Federation of Gynecology and Obstetrics (FIGO). The average age was 56.2 years. Fifty of the 62 women had low-grade EEC (Grades 1 and 2) and 12 were high-grade (Grade 3). Fifty-two patients presented with early-stage tumors (Stage IA to II) and 10 presented with advanced-stage tumors (IIIA to IVB). Sixty control normal endometrial tissues from females without EEC (mean age 53.1 years) were obtained. Thirty-four were in the proliferative phase and 26 were in the secretory phase. All the formalin-fixed, paraffin-embedded sections were obtained from the files of the Department of Pathology of China-Japan Friendship Hospital and Beijing Chao Yang Hospital of Capital Medical University. The study was approved by the ethics committee of China-Japan Friendship Hospital, Beijing, China. No initial hormonal therapy or radiotherapy was performed prior to endometrium excision. Hematoxylin-eosin (H&E) stained sections from each case were reviewed and representative sections from each tumor were selected.

### Immunohistochemical analysis of claudin-4 expression in endometrial carcinoma

Immunohistochemical stains were performed on 5-*μ*m-thick sections of formalin-fixed, paraffin-embedded tissues. After antigen retrieval, the sections were incubated with monoclonal mouse anti-claudin-4 (Zymed, San Francisco, CA, USA). Antigen-bound primary antibody was detected using standard avidin-biotin immunoperoxidase complex (DAKO Corp., Carpinteria, CA, USA). Negative controls, in which the primary antibodies were not added, were processed in parallel. In each case, two independent observers recorded the distribution of staining, intensity and localization. Cases were classified as follows regarding the intensity of protein expression: −, no immunostaining present; +, weak staining; ++, medium staining and +++, intense staining.

### Real-time PCR analysis of claudin-4 expression in endometrial carcinoma

RNA isolation was performed using TRIzol reagent (Sangon, Shanghai, China) according to the manufacturer’s instructions. Total RNA (5 *μ*g) from each sample was reverse transcribed using M-MLV reverse transcriptase (Promega Corp., Madison, WI, USA). The SYBR-Green I assay was used for detecting real-time PCR products of claudin-4. The primers used were as follows: claudin-4 (forward, 5′-GTGCCTTGCTCACCGAAAC; reverse, 5′-CCACCACTGCCCAAACCT) and glyceraldehyde phosphate dehydrogenase (GAPDH) (forward, 5′-GAAGATGGTGATGGGATTTC; reverse, 5′-GAAGGT GAAGGTCGGAGT). A four-step experimental protocol was used: i) denaturation program (10 min at 95°C); ii) amplification and quantification program repeated 40 times (10 sec at 95°C; 5 sec at 57°C for claudin-4 or 5 sec at 55°C for GAPDH; 10 sec at 72°C for claudin-4 or 15 sec at 72°C for GAPDH with a single fluorescence measurement); iii) melting curve program (65–95°C with a heating rate of 0.1°C/sec and a continuous fluorescence measurement); iv) cooling program down to 40°C. Specificity of the amplified PCR product was assessed by performing melting curve analysis. The relative expression is based on the expression ratio of claudin-4 versus GAPDH.

### Cell culture

The human endometrial carcinoma cell line Ishikawa (a high expresser of claudin-4; data not shown) was a generous gift from Professor Li-Hui Wei (Peking University People’s Hospital, Beijing, China) and was obtained from American Type Culture Collection (Manassas, VA, USA). The Ishikawa cell line was established from a well-differentiated human endometrial carcinoma. Cells were cultured in RPMI-1640 medium supplemented with 10% fetal bovine serum (FBS) plus penicillin and streptomycin. They were subcultured when the density reached 80% using 0.25% trypsin in Ca^2+^/Mg^2+^-free PBS. Cell viability was determined by trypan blue exclusion. Cells were brought to a density of 1×10^7^ cells/ml for injection. Harvested cells with >95% viability by trypan blue exclusion were considered acceptable for injection.

### In vivo antitumor effect of cisplatin and paclitaxel against Ishikawa endometrial carcinoma cells

Ninety female BALB/c nu/nu mice (5 weeks old) were obtained from the Zoology Institute of the Chinese Academy of Sciences (Beijing, China) and housed in a pathogen-free environment. All experiments were approved by the Institute’s Animal Care and Use Committee. For subcutaneous xenografts, 1×10^7^ viable Ishikawa cells were injected subcutaneously into the right flank. After 7 days, when established tumors of ∼6 mm in diameter were detectable, mice were randomized in groups (n=30) to receive different treatments. One group was treated intraperitoneally with cisplatin (5 mg/kg/day on days 8, 15 and 22). Paclitaxel was used as reference compound (30 mg/kg/day on days 8, 15 and 22). Control mice were injected with 0.9% saline solution. All mice were sacrificed on the 30th day after tumor implantation. The final weight for each animal was measured just prior to sacrifice. The tumors were excised from the mice, weighed for the calculation of mean tumor weight for every group and prepared for subsequent claudin-4 immunohistochemical staining and mRNA analysis. The tumor dimensions were measured with calipers and the tumor volume was calculated using the formula: 0.52 × larger diameter × (smaller diameter)^2^.

### Immunohistochemical and real-time PCR analysis of claudin-4 expression in tumor xenografts

Immunohistochemical and real-time PCR analysis of claudin-4 expression in tumor xenografts was performed using the method described above.

### Statistical analysis

The statistical significance of the data was evaluated using one-way ANOVA and Chi-square analysis. P<0.05 was considered to indicate a statistically significant result. All statistical analysis was calculated using SPSS 11.0 (SPSS, Inc, Chicago, IL, USA).

## Results

### Immunohistochemical expression of claudin-4 in EEC

**Figure 1:**
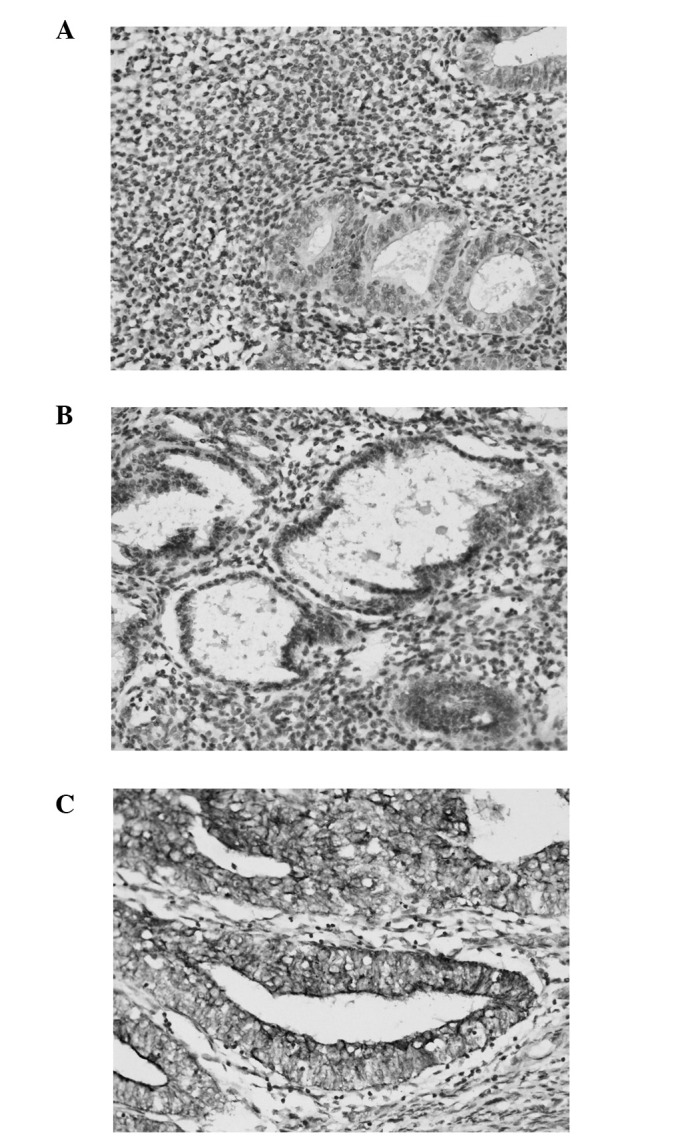
Representative immunohistochemical micrographs of claudin-4 in endometrial carcinoma sections (original magnification, ×400). (A) Normal proliferative phase endometrium. (B) Normal secretory phase endometrium. (C) Endometrioid endometrial carcinoma.

The immunohistochemical analysis of claudin-4 showed a specific brownish immunostaining localized to the glandular epithelial cell membrane. There was no signal detected in the stromal cell. All EEC samples demonstrated some degree of claudin-4 expression. Glandular epithelial cells in EEC exhibited a circumferential membranous pattern of staining for claudin-4. Among the EEC samples, 21/62 (33.9%) showed medium staining for claudin-4 and 41/62 (66.1%) showed intense staining for claudin-4. Of the normal endometrial tissue, 28/60 (46.7%) showed weak staining and 32/60 (53.3%) showed no staining for claudin-4. There was a statistically significant difference in claudin-4 expression between EEC and normal endometrial tissue. Claudin-4 immunostaining was stronger and more diffuse in EEC than in normal cyclic endometrium (Fig. 1).

### Expression of claudin-4 mRNA

According to real-time PCR, the relative quantity of claudin-4 was 169.7±11.8 in the EEC group and 17.9±3.2 in normal endometrium. Claudin-4 was found to be highly upregulated in EEC (P<0.01), consistent with the result obtained using immunohistochemistry.

### Claudin-4 expression in Ishikawa xenografts after treatment with cytotoxic drugs

A total of 88 out of 90 animals survived treatment with cisplatin. Statistically significant body weight change was not found with paclitaxel administration and no significant change in tumor volume was demonstrated in the paclitaxel group compared with controls. A significant reduction in tumor growth and weight loss occurred with cisplatin compared with the group treated with paclitaxel (Table I).

**Figure 2:**
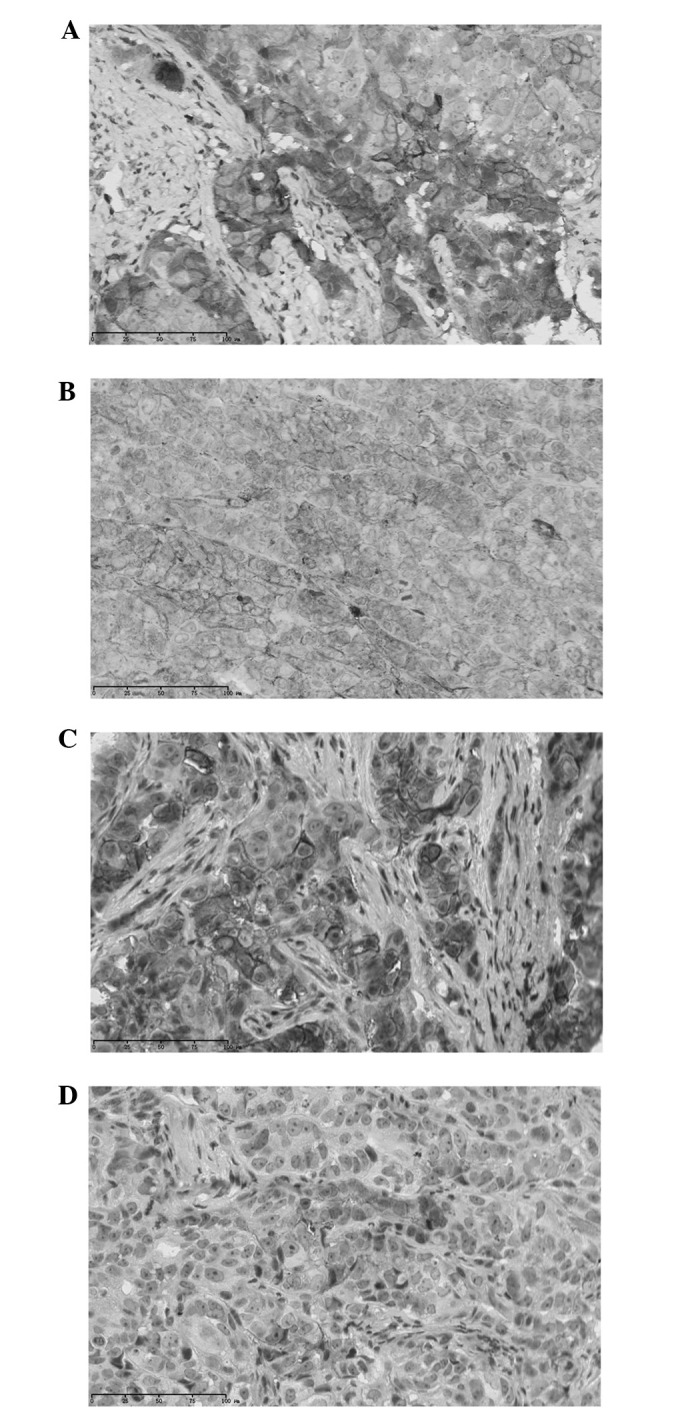
Representative immunohistochemical micrographs of claudin-4 in Ishikawa xenograft sections (original magnification, ×400). (A) Before treatment with paclitaxel. (B) After treatment with paclitaxel. (C) Before treatment with cisplatin. (D) After treatment with cisplatin.

After treatment with cytotoxic drugs, claudin-4 expression in Ishikawa xenografts was detected using immunohistochemical stain. Claudin-4 was 100% positive in the control group, and generally had a membranous staining pattern. A similar result was found in the paclitaxel group, 30 (100%) of 30 cases were positive for claudin-4, with 18 cases showing +++ staining, 10 showing ++ staining and 2 showing + staining. A significant decrease in claudin-4 expression was observed in the cisplatin group, with 4 cases showing +++ staining, 18 showing ++ staining and 6 showing + staining (Table I, Fig. 2).

To get highly sensitive measurement of claudin-4 at the transcript level, a real-time PCR assay was developed. Before treatment with cytotoxic drugs, the expression levels of claudin-4 in the cisplatin, paclitaxel and control group were 273.1±26.8, 284.7±30.1 and 279.8±28.6, respectively. There was no difference among the three groups. After treatment with cytotoxic drugs, the relative quantity of claudin-4 in the three groups was 153.4±34.7, 248.9±28.4 and 262.1±25.9, respectively. Claudin-4 mRNA in the control group was slightly higher than that in paclitaxel group, but there was no significant statistical difference. The cisplatin group showed a significant decrease in mRNA level of claudin-4 compared with the paclitaxel and control group, consistent with the result obtained using immunohistochemical analysis.

## Discussion

Claudins are the major integral membrane proteins forming the backbone of tight junctions. Increased claudin-4 levels have been shown in prostate cancer (9), ovarian carcinoma (10) and in several other tumor cell lines (11). On the basis of these observations, claudin-4 may represent a useful biomarker for detection and diagnosis of certain cancers (12). The exact role of claudin-4 overexpression and the functional importance of claudin-4 in the development of cancer remain unclear.

Recently, Santin *et al*(13) used gene expression studies to demonstrate that claudin-4 is among the highest upregulated genes in USPC when compared with normal endometrial cells. In this study the expression of claudin-4 was much higher in EEC than in normal cyclic endometrium. Although EEC and USPC belong to two different pathogenetic types of endometrial carcinoma, these results have led to the suggestion that upregulated claudin-4 may be involved in endometrial carcinogenesis.

It had been shown that cisplatin and paclitaxel had an anti-tumor effect on human cancer xenografted nude mice (14,15). To investigate the role of claudin-4 as a marker for the activity of anti-neoplastic agents the same model was used. In the present study, immunohistochemical and PCR analysis of tumor xenografts demonstrated that cisplatin treatment is capable of producing a significant decrease in claudin-4 expression which correlated with the reduction in tumor volume. The correlation between the expression of claudin-4 and the change in tumor volume due to chemotherapy demonstrates that therapeutic inhibition of claudin-4 may reduce claudin-expressing cells and it further suggests a role of claudin-4 as a marker for the activity of anti-neoplastic agents.

The antitumor activity of paclitaxel against solid tumors, such as breast and ovarian cancer, has been well established (16,17). No significant changes in tumor volume and claudin-4 expression were shown in the paclitaxel group in this study, however. The route of administration and dose level may limit the effect of paclitaxel. Cisplatin seemed to be an effective anti-neoplastic agent, exhibiting a significantly higher cytotoxic effect than paclitaxel. Two mice died and a significant weight loss occurred among the cisplatin-treated animals. The development of innovative, effective therapy against endometrial cancer remains a high priority.

Notably, claudin-4 is a receptor for *clostridium perfringens* enterotoxin (CPE). Experiments have shown that CPE has a cytotoxic effect towards cancer cells, provided these cells express claudin-4 (18–20). Further studies are required to demonstrate that CPE holds promise for the development of alternative anticancer agents for endometrial carcinoma.

In conclusion, this study was undertaken to understand the biological significance of altered claudin-4 expression in endometrial carcinoma. Other mechanisms relevant to claudin-4 overexpression in endometrial cancer are still under investigation. The present observations raise the possibility of exploiting claudin-4 as a potential biomarker for endometrial carcinoma and may provide an opportunity for therapeutic intervention.

## Figures and Tables

**Table I:** Claudin-4 expression in Ishikawa xenografts after treatment with cytotoxic drugs.

				Claudin-4 expression, n (%)			
Treatment	n	Body weight (g)	Tumor volume (cm^3^)	−	+	++	+++
Control	30	19.32±1.54	1.22±0.13	0	0	10 (33.3)	20 (66.7)
Cisplatin^a^	28	0.71±2.23	0.79±0.27	0	6 (21.4)	18 (64.3)	4 (14.3)
Paclitaxel	30	18.16±2.89	1.14±0.18	0	2 (6.7)	10 (33.3)	18 (60.0)

Values are the mean ± SD.

a P<0.05 compared with control and paclitaxel group.
